# Seeking the Light in Intensive Care Unit Sedation: The Optimal Sedation Strategy for Critically Ill Patients

**DOI:** 10.3389/fmed.2022.901343

**Published:** 2022-06-24

**Authors:** Nicola Gitti, Stefania Renzi, Mattia Marchesi, Michele Bertoni, Francisco A. Lobo, Frank A. Rasulo, Alberto Goffi, Matteo Pozzi, Simone Piva

**Affiliations:** ^1^Department of Medical and Surgical Specialties, Radiological Science and Public Health, University of Brescia, Brescia, Italy; ^2^Department of Anesthesia, Critical Care and Emergency, Spedali Civili University Hospital, Brescia, Italy; ^3^Institute of Anesthesiology, Cleveland Clinic Abu Dhabi, Abu Dhabi, United Arab Emirates; ^4^Interdepartmental Division of Critical Care Medicine, Department of Medicine, University of Toronto, Toronto, ON, Canada; ^5^Department of Emergency and Intensive Care, San Gerardo Hospital, Monza, Italy

**Keywords:** ICU—intensive care unit, neuromonitoring, light sedation, dexmedetomedine, propofol

## Abstract

The clinical approach to sedation in critically ill patients has changed dramatically over the last two decades, moving to a regimen of light or non-sedation associated with adequate analgesia to guarantee the patient’s comfort, active interaction with the environment and family, and early mobilization and assessment of delirium. Although deep sedation (DS) may still be necessary for certain clinical scenarios, it should be limited to strict indications, such as mechanically ventilated patients with Acute Respiratory Distress Syndrome (ARDS), status epilepticus, intracranial hypertension, or those requiring target temperature management. DS, if not indicated, is associated with prolonged duration of mechanical ventilation and ICU stay, and increased mortality. Therefore, continuous monitoring of the level of sedation, especially when associated with the raw EEG data, is important to avoid unnecessary oversedation and to convert a DS strategy to light sedation as soon as possible. The approach to the management of critically ill patients is multidimensional, so targeted sedation should be considered in the context of the ABCDEF bundle, a holistic patient approach. Sedation may interfere with early mobilization and family engagement and may have an impact on delirium assessment and risk. If adequately applied, the ABCDEF bundle allows for a patient-centered, multidimensional, and multi-professional ICU care model to be achieved, with a positive impact on appropriate sedation and patient comfort, along with other important determinants of long-term patient outcomes.

## Introduction

Patients admitted to the intensive care unit (ICU) routinely experience pain, agitation and anxiety, use of invasive monitoring, and need for invasive procedures or mechanical ventilation. Appropriate analgesia and sedation are therefore essential. In mechanically ventilated patients, sedation aims to minimize oxygen consumption, keep patients comfortably connected to the ventilator, avoid recall of the critical condition and prevent psychological and physical damage. Failure to control pain and agitation can have detrimental effects both in the short- and long-term perspective. Poorly controlled pain and agitation have been associated with worsening of the critical condition, increase in sympathetic tone, and increased rate of accidental removal of medical devices such as endotracheal tubes and vascular catheters (1–3). Moreover, uncontrolled pain during ICU stay is related to a higher incidence of chronic pain, post-traumatic stress disorder (PTSD) symptoms, and a lower health-related quality of life (HRQoL). Conversely, deep and prolonged sedation has been associated with increased duration of mechanical ventilation, delayed weaning, increased incidence of delirium, and muscle impairment leading to ICU-acquired weakness (ICU–AW), and higher hospital and 6-month mortality (1). Different studies have been conducted in the last two decades to clarify the “why” and “how” to sedate patients in ICU. The key concept is that a “calm, comfortable, and collaborative” patient (i.e., lightly sedated) allows active cognitive stimulation, earlier liberation from the endotracheal tube, active mobilization, and also improved interaction with the healthcare team and the family, all of them being important patient-centered outcomes.

## The “Why”

The paradigm of sedation in ICU changed dramatically in the last few decades. In the 80s, the common belief was that patients should adapt to machines, leading to the large use of deep sedation (DS) and prolonged immobility. In the 90s, the paradigm changed: the machine should adapt to the patients but clinicians should avoid patients’ recall and memory of critical ill, leading to the use of deep sedation and avoiding neuromuscular blocking agent (NMBA). Starting from the early twentieth century, the idea of a protocolized sedation with a daily sedation interruption (DSI) modified dramatically the sedation approach in mechanically ventilated patients. A protocolized sedation approach alone (intended as sedation delivered by team members following written and approved procedures and outlined in a protocol) is not clearly associated with a beneficial effect in terms of duration of mechanical ventilation, mortality, or ICU length of stay as highlighted by a recent Cochrane systematic review (SR) and meta-analysis (MA) review, and confirmed in a recent multicenter cluster-RCT (DESIST trial) (Table 1) (4, 5). Indeed, the association between sedation protocol and daily sedation interruption (DSI) was initially introduced by Kress et al. (6) leading to a large number of trials aiming to establish its efficacy and safety (Table 1). Daily sedation interruption (DSI) seems to reduce time in coma, ICU and hospital length of stay (LOS), sedation time, and mechanical ventilation duration and improve the 1-year survival rates (7). Although other trials did not find the same results (8, 9), subsequent systematic reviews (SRs) and meta-analysis (MA) of RCTs demonstrated a possible reduction in duration of mechanical ventilation, ICU–LOS, hospital-LOS, and also a reduction in tracheostomy rate (Table 1). On the other hand, the main arguments against daily sedation interruption (DSI) consist in its side effects, including agitation, pain, and endotracheal tube or catheter self-removal. Although possible psychological sequelae, such as anxiety, depression, and PTSD, related to the patient’s awareness of their critical situations are claimed against daily sedation interruption (DSI), different studies demonstrated that such an approach did not impact the recall of ICU stay nor increased the incidence of PTSD (10, 11). Daily sedation interruption (DSI) could increase nurse overload, especially in resources limited counties where the nurse: patients ratio is frequently less than 1:1, and it should be accompanied by a light sedation (LS) approach for the rest of the day, instead of a deep sedation approach (12). Indeed, no doubts exist about the impact of deep sedation during the first 48 h of ICU and later in ICU stay, as demonstrated by Shehabi et al. in the two different studies (13, 14). Deep sedation (DS) was an independent predictor of long-term mortality and time to extubation in mechanically ventilated ICU patients. Although early deep sedation and the cumulative dose of sedative agents were not associated with time to delirium after 48 h, patients with lighter sedation had a lower presence of delirium at 48 h, and also significantly more coma- and delirium-free days at 28 days. These results induced the authors to propose and test the so-called “early goal-directed sedation” concept (i.e., light sedation, LS) in a pilot RCT (15). Starting early after mechanical ventilation (MV) initiation, this approach refers to goal-directed management to target a light level of sedation and minimize benzodiazepine usage. Results showed that patients with light sedation received less benzodiazepine and propofol, had more delirium-free days and required fewer physical restraints, but mechanical ventilation (MV) duration, ICU, and hospital LOS or mortality were not modified. Starting from these contrasting results, the effects of light sedation have been examined in different subsequent RCTs summarized in SRs and MAs (Table 1). Among the most important systematic reviews, Stephens et al. showed as the implementation of light sedation in the first 48 h of mechanical ventilation reduced mortality, mechanical ventilation duration, and ICU–LOS (16). The effects of light sedation on outcomes were also examined in the meta-analysis presented in the PADIS guidelines, showing a reduction in mechanical ventilation (MV) duration and the tracheostomy rate, with no effects on 90-day mortality, the occurrence of delirium, depression, PTDS, or self-extubation (12). The heterogeneity between included RCTs and their low quality forced the guidelines panel to make use of light sedation as a conditional recommendation. At last, Aitken et al. (17) examined the effect of depth of sedation in ICU patients on outcomes that extend across the ICU stay and beyond, including 7 RCTs and 18 cohort studies. The main findings were that lighter sedation was not associated with the reduced ICU or hospital mortality. Moreover, lighter sedation was not associated with a reduced duration of mechanical ventilation, delirium occurrence, and other adverse events among the RCTs but it was among the cohort studies. Across studies, both risks of bias and heterogeneity were high. The different results found in the Aitken and PADIS MAs are difficult to explain, although the inclusion of non-RCTs in Aitken’s study inevitably introduced heterogeneity in the results, the RCTs included are different, and Aitken’s study included the results from the NON-SEDA trial (18) that account for 45% of the patients included in the MA. NON-SEDA trial (18) is the far biggest RCT that investigated the effects of a non-sedation protocol compared with the light sedation, and the authors did not find any differences in terms of 90-day mortality, ventilator-free days, ICU and hospital LOS. A possible explanation for the non-superiority of a non-sedation strategy in the RCT, as the authors stated in the discussion, could be that the depth of sedation did not differ between the groups as much as intended, especially on day 1, configuring the non-sedation approach as light sedation (mean RASS score was −2.3 on day 1 in light sedation vs. −1.3 in the non-sedation group) (18).

**TABLE 1:** Summary of studies mentioned in the review.

Title	Study type	References	Outcomes assessed	Results	Year
**Protocol-directed sedation (PDS)**					
Protocol-directed sedation vs. non-protocol-directed sedation to reduce duration of mechanically ventilated intensive care patients.	SR- MA	Aitken et al. (4)	Duration of MV ICU mortality Hospital mortality ICU LOS Hospital LOS Incidence of self extubation Incidence of reintubation Incidence of tracheostomy	Unchanged Unchanged Unchanged Unchanged Reduced with PDS Unchanged Unchanged Unchanged	2015
Staff education, regular sedation and analgesia quality feedback, and a sedation monitoring technology for improving sedation and analgesia quality for critically ill, mechanically ventilated patients: a cluster randomized trial.	Cluster- RCT	Walsh et al. (5)	ICU mortality Hospital mortality Time to cessation of mechanical ventilation Time to discharge from ICU Time to discharge from hospital	Unchanged Unchanged Unchanged Unchanged Unchanged	2016
**Daily sedation interruption (DSI)**					
Sedation of critically ill patients during mechanical ventilation. A comparison of propofol and midazolam.	RCT	Kress et al. (6)	Time to awaken Time to sedation Reproducibility of bedside assessment of sedation’s level Change in VO2 from awake to sedated state	Decreased in Propofol-group Unchanged Unchanged Decreased similarly in sedated state	1996
Efficacy and safety of a paired sedation and ventilator weaning protocol for mechanically ventilated patients in intensive care (Awakening and Breathing Controlled trial): a randomized controlled trial	RCT	Girard et al. (7)	Ventilator free days ICU LOS Hospital LOS 28-day mortality 1-year mortality Days of coma Days of delirium RASS at first SBT Any self extubation Self-extubation requiring reintubation Reintubation Tracheostomy	Increased Decreased Decreased Unchanged Increased Decreased Unchanged Decreased Increased Unchanged Unchanged Unchanged	2008
Daily sedation interruption in mechanically ventilated critically ill patients cared for with a sedation protocol: a randomized controlled trial	RCT	Mehta et al. (8)	Days to successful extubation ICU LOS Hospital LOS ICU mortality Hospital mortality Unintentional device removal Neuroimaging in ICU Physical restraint Delirium Tracheostomy Nurse workload	Unchanged Unchanged Unchanged Unchanged Unchanged Unchanged Unchanged Unchanged Unchanged Unchanged Increased in PS + DSI	2012
Daily sedation interruption vs. intermittent sedation in mechanically ventilated critically ill patients: a randomized trial	RCT	Nassar and Park (9)	Ventilator-free days in 28 days ICU mortality Hospital mortality Incidence of Delirium Delirium or coma-free days Median SAS Percentage of time on target SAS ICU LOS Hospital LOS Reintubation Self-extubation Accidental removal of catheters Tracheostomy	Unchanged Unchanged Unchanged Unchanged Unchanged Decreased in DSI group Unchanged Unchanged Unchanged Increased in DSI group Increased in DSI group Unchanged Unchanged	2014
The long-term psychological effects of daily sedative interruption on critically ill patients	RCT	Kress et al. (11)	Duration of mechanical ventilation ICU LOS Incidence of new medical illness Recall awakening in the ICU Total impact of Events score Avoidance subscale score Intrusive Thoughts subscale score Diagnosis of PTSD SF-36 score Chronic Anxiety levels Acute Anxiety levels Beck Depression Index score PAIS T-score	Unchanged Unchanged Unchanged Reduced Reduced Reduced Reduced Unchanged Unchanged Unchanged Unchanged Unchanged Unchanged	2003
Meta-analysis of randomized controlled trials on daily sedation interruption for critically ill adult patients	SR-MA	Augustes et al. (54)	Duration of mechanical Ventilation Risk of OT tube removal Tracheostomy Re-Intubation Rate ICU LOS and H-LOS Mortality	Unchanged Unchanged Reduced Unchanged Unchanged Unchanged	2011
Effects of daily sedation interruption in intensive care unit patients undergoing mechanical ventilation: A meta-analysis of randomized controlled trials	MA	Chen et al. (55)	Duration of mechanical ventilation ICU-LOS sedation Duration HLOS Re-intubation Rate Unplanned device removal Tracheostomy risk Ventilator-associated pneumonia risk ICU mortality	Reduced Reduced Reduced Reduced Unchanged Unchanged Unchanged Unchanged	2021
Daily sedation interruption vs. no daily sedation interruption for critically ill adult patients requiring invasive mechanical ventilation	SR	Burry et al. (56)	Duration of mechanical ventilation ICU LOS HLOS Mortality OT Tube removal Catheter removal Delirium QoL Drugs Dosage Tracheostomy	Unchanged Unchanged Unchanged Unchanged Unchanged Unchanged Unchanged Unchanged Unchanged Reduced	2014
Effectiveness of daily interruption of sedation in sedated patients with mechanical ventilation in ICU: A systematic review	SR	Chen et al. (57)	ICU-LOS Duration of mechanical Ventilation OT tube removal Tracheostomy	Reduced Reduced Unchanged Reduced	2014
**Deep sedation vs. light sedation (DS vs. LS)**					
Early goal-directed sedation vs. standard sedation in mechanically ventilated critically ill patients: a pilot study	RCT	Shehabi et al. (15)	Time with RASS −2 to −1 first 48 h Time with RASS −3 to −5 first 48 h Dexmedetomidine received Midazolam received Propofol received Morphine received Fentanyl received CAM-ICU + ve Days with −ve CAM-ICU Mobilization Neuromuscular blockade Physical restraint Extubated within 7 days Device removal or self-extubation Ventilator-free days at day 28 ICU LOS Hospital LOS Hospital mortality 90-day mortality	Increased Decreased Increased Decreased Decreased Unchanged Unchanged Unchanged Unchanged Unchanged Unchanged Decreased Increased Unchanged Unchanged Unchanged Unchanged Unchanged Unchanged	2013
Practice patterns and outcomes associated with early sedation depth in mechanically ventilated patients: A systematic review and meta-analysis	SR-MA	Stephens et al. (16)	Hospital mortality rate Delirium incidence Tracheostomy incidence Mechanical ventilation days ICU LOS Hospital LOS	Decreased in Early light sedation group Unchanged Unchanged Reduced in Early light sedation group Reduced in Early light sedation group Unchanged	2018
PADIS					
Inconsistent relationship between depth of sedation and intensive care outcome: systematic review and meta-analysis	SR-MA	Aitken et al. (17)	ICU mortality (RCTs) Mechanical ventilation duration (RCTs) Mechanical ventilation duration (Cohort studies) TIme to extubation (Cohort studies) ICU LOS (Cohort studies) Hospital LOS (Cohort studies) Ventilator associated pneumonia (Cohort studies) Hospital mortality (RCTs and Cohort studies) Delirium (RCTs and Cohort studies) Adverse events (RCTs and cohort studies)	Unchanged Unchanged Reduced in light sedation group Reduced in light sedation group Reduced in light sedation group Reduced in light sedation group Reduced in light sedation group Unchanged Unchanged Unchanged	2021
Non-sedation or Light Sedation in Critically ill, Mechanically Ventilated Patients	RCT	Olsen et al. (18)	Mortality at 90 days No. of days until death up to 90 days No. of major thromboembolic events No. of days free from coma or delirium within 28 days Highest measured RIFLE score within 28 days No. of ICU-free days No. of ventilator free days	Unchanged Unchanged Unchanged Unchanged Unchanged Unchanged Unchanged	2020
**SEDATIVE DRUGS**					
Effect of sedation with dexmedetomidine vs. lorazepam on acute brain dysfunction in mechanically ventilated patients: the MENDS randomized controlled trial	RCT	Pandharipande et al. (28)	Delirium-free and coma-free days Delirium-free days Coma-free days Prevalence of delirium or coma Prevalence of delirium Prevalence of coma Mechanical ventilator-free Intensive care unit length of stay 28-day mortality	Increased in dex group Unchanged Decreased in dex group Decreased in dex group Unchanged Decreased in dex group Unchanged Unchanged Unchanged	2007
Dexmedetomidine vs. midazolam or propofol for sedation during prolonged mechanical ventilation: two randomized controlled trials	RCT	Jakob et al. (29)	Time of RASS range between 0 and −3 without rescue therapy MIDEX PRODEX Duration of mechanical ventilation MIDEX PRODEX Nurses’ assessment of VAS MIDEX PRODEX ICU LOS MIDEX PRODEX	Unchanged Unchanged Reduced in dex group Unchanged Increased in dex group Increased in dex group Unchanged Unchanged	2012
Early sedation with dexmedetomidine in ventilated critically ill patients and heterogeneity of treatment effect in the SPICE III randomized controlled trial	Cluster-RCT	Shehabi et al. (36)	90-day mortality >65 years ≤65 years Cluster 1 (operative diagnosis) Cluster 2 (non-operative diagnosis) Coma and delirium free days >65 years <65 years Cluster 1 (operative diagnosis) Cluster 2 (non-operative diagnosis) Ventilator free days >65 years <65 years Cluster 1 (operative diagnosis) Cluster 2 (non-operative diagnosis)	Decreased Increased Decreased Increased Increased Decreased Increased Increased Increased Decreased Increased Increased	2021
Effect of dexmedetomidine vs. lorazepam on outcome in patients with sepsis: an *a priori*-designed analysis of the MENDS randomized controlled trial	RCT	Pandharipande et al. (40)	Septic patients: Delirium/coma-free days Delirium-free days Coma-free days MV-free days ICU days 28-day mortality Non-septic patients: Delirium/coma-free days Delirium-free days Com-free days MV-free days ICU days 28-day mortality	Increased Unchanged Increased Increased Unchanged Decreased Unchanged Unchanged Unchanged Unchanged Unchanged Unchanged	2010
Dexmedetomidine or Propofol for Sedation in Mechanically Ventilated Adults with Sepsis	RCT	Hughes et al. (42)	Days alive without delirium or coma at 14 days Ventilator-free days at 28 days Death at 90-days GLobal cognition	Unchanged Unchanged Unchanged Unchanged	2021
**Neuromonitoring**					
BIS monitoring vs. clinical assessment for sedation in mechanically ventilated adults in the intensive care unit and its impact on clinical outcomes and resource utilization	SR-MA	Shetty et al. (48)	ICU LOS Duration of Mechanical ventilation Risk of adverse events Amount of sedative agents administered	Unchanged (low quality evidence) Unchanged (low quality evidence) Unchanged (very low quality evidence) Not assessable	2018

**FIGURE 1:**
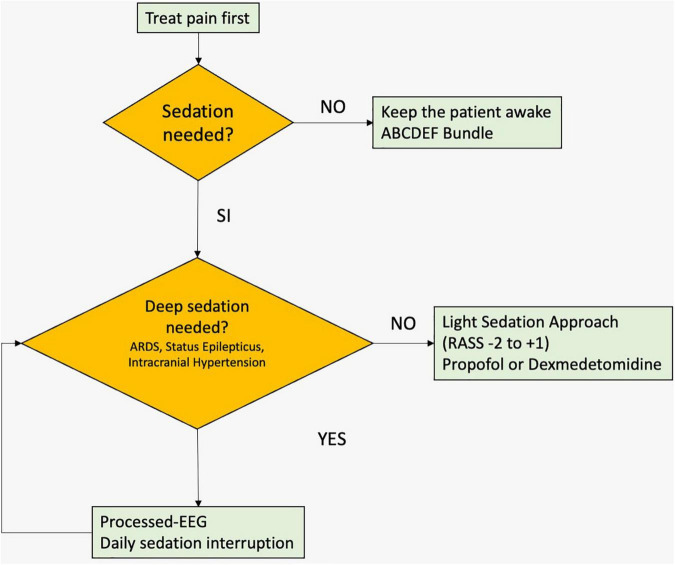
Algorithm for the use of sedation in critically ill patients. ARDS, acute respiratory distress syndrome; RASS, Richmond Agitation–Sedation Scale.

Although still under debate, light sedation has been suggested by the 2018 Pain, Agitation/Sedation, Delirium, Immobility, and Sleep Disruption (PADIS) guidelines (12), and reinforced by the eCASH approach (19). In particular, a protocol based, stepwise assessment for pain control and sedation management in critically ill patients should be adopted, and light sedation “should be used in all mechanically ventilated patients” (conditional recommendation, low quality of evidence) (Figure 1).

Some caveats exist in the application of light sedation. At first, its definition is not universally accepted. The PADIS guidelines define light sedation as a Richmond Agitation Sedation Scale (RASS) score between + 1 (slightly restless) and −2 (awake with eye contact to voice) or a Riker Sedation–Agitation Scale (SAS) score between 4 (calm and cooperative) and 3 (difficult to rouse and obey simple commands). The SAS does not evaluate arousal and an SAS of 3 points is the only score to assign to a sedated patient without coma (SAS = 1 or 2) (20). The RASS scale may have a positive or negative value (−5 to + 4) and it is an ordinal scale, which makes it unuseful to synthesize using an average score (21). This is particularly important since the sedation level could variate during the 24 h; for this reason, other methods for sedation assessment have been proposed, such as the Sedation Index (22), which summarizes the sedation level over 48 h (sum of the negative RASS scores (RASS −1 to −5) divided by the total number of RASS measurements performed). When Sedation Index has been used, the level of sedation was strongly related to the risk of death, delirium, and delayed time to extubation (22).

The second caveat is that when light sedation is not integrated into a bundle of patient-centered outcomes, could not be as useful. The eCASH–early Comfort using Analgesia, minimal Sedatives and maximal Humane care–as well as the ABCDEF-R bundle (where R = respiratory-drive-control has been proposed recently) (12, 23), suggest the integration of light sedation into a bundle to avoid pain, anxiety, agitation, delirium, and immobility, to reduce the post-intensive care syndrome (PICS) incidence. These targets could be achieved by treating pain first and by accompanying light sedation to communications aids, noise reduction to promote sleep, early mobilization, delirium monitoring, and family involvement.

Occasionally, deep sedation may be required. In the case of patients with ARDS, historically deep sedation along with NMBA continuous infusion has been considered the best clinical practice (24). Following recent evidence (25), practical clinical guidelines (26) suggest avoiding routine use of NMBA infusion in adults with ARDS before optimizing mechanical ventilation and assessing ARDS severity. Moreover, the authors suggest against using an NMBA infusion (Suggestion, low certainty of evidence) in patients who tolerate ventilation using a lighter sedation strategy; whenever an NMBA is required to facilitate lung-protective ventilation, it should be used intermittently (Suggestion, low certainty of evidence) (26).

## The “How”

When the “why” of sedation in ICU has been explored, clinicians want to know “how” to lightly sedate the patients. On this topic, there is no doubt that benzodiazepines should be avoided to minimize the risk of delirium (27–33). As a result, dexmedetomidine and propofol remain the possible choices.

Dexmedetomidine is a highly selective α2 adrenergic receptor antagonist that produces dose-dependent sedation with no respiratory depression and modest hemodynamic effects (34). Dexmedetomidine may promote sleep *via* more physiological pathways in comparison with GABAergic sedatives (i.e., benzodiazepines, propofol), favoring the N3 (or slow wave sleep) stage (35). Propofol is a gamma-aminobutyric acid (GABA) receptor agonist, by binding to the β subunit of the postsynaptic GABA–A receptor, it induces postsynaptic membrane hyperpolarization and inhibits neuronal depolarization.

Pain, Agitation/Sedation, Delirium, Immobility, and Sleep Disruption guidelines recommend the use of propofol or dexmedetomidine over the benzodiazepine for sedation in critically ill, mechanically ventilated adults, as a conditional recommendation with low quality of evidence (12). Different RCTs have investigated the effects of dexmedetomidine as sedative agents, only one of them compared dexmedetomidine with propofol (29). The SPICE III trial is the most recent and biggest RCT on this topic, including approximately 8 times as many patients as the other trials (36). It compared the use of dexmedetomidine with usual care (propofol or midazolam or a combination of the two) for sedation therapy in mechanically ventilated patients. This open-label, randomized trial enrolled 3,904 patients, with mortality at 90 days as the primary outcome. Secondary outcomes included mortality, cognitive function, and HRQoL assessed at 180 days. Results from the study revealed no difference in 90- and 180-day mortality, nor in cognitive function and HRQoL (17).

The explanation for such unexpected results should be found in the high proportion of patients (60% on day 1 and 50% on day 2) who required deep sedation (not allowed in all the other trials), which may have independently influenced outcomes, as discovered previously by the same authors (14, 22). Moreover, more than 70% of patients in the dexmedetomidine arm received propofol or midazolam to reach goal sedation in the first 48 h following randomization, and more than 50% of the dexmedetomidine cohort continued to receive propofol at the study day 10 (with only 30% of patients requiring deep sedation). The inadequacy of dexmedetomidine to maintain light sedation has been reported also in MIDEX (29) and PRODEX (29) trials, with, respectively, 43.8 and 72.5% of patients requiring rescue sedation in the dexmedetomidine arm. Another interesting result of the SPICE III trial is the higher rate of adverse events in the dexmedetomidine cohort compared with the usual care cohort (9.6 vs. 1.8%, respectively, *P* < 0.0001), although lower than in the other trials.

A subsequent Bayesian secondary analysis of the SPICE III trial (36) found that dexmedetomidine exhibited a high probability of reduced 90-day mortality in older patients (more than 65 years) and a high probability of increased 90-day mortality in the younger patients of non-operative status.

An important topic is the cost of sedation. Park and Jeong (37) carried out a cost-minimization analysis based on MIDEX and PRODEX studies, demonstrating that patients on a mechanical ventilator and treated with midazolam and propofol had medical costs (related drug costs, ICU care costs, and costs of treating adverse events), respectively, 17.6 and 15.2% more than patients treated with dexmedetomidine. A secondary analysis of the SEDCOM trial (27) (an RCT comparing dexmedetomidine and midazolam for sedation in critically ill patients) (38) showed a median total intensive care unit cost savings of $9,679 using dexmedetomidine in patients ventilated for more than 24 h. The primary cost drivers were reduced costs of ICU stay and reduced costs of mechanical ventilation. Aggarwal et al. (39) conducted a cost-minimization analysis for short sedation in ICU, demonstrating that dexmedetomidine was associated with significant cost savings of ∼$6,000 compared with propofol and midazolam in mechanically ventilated, adult patients undergoing short-term sedation (<24 h). Although the limited generalizability of cost data outside the US, the cost-saving was mainly related to the reduced ICU length of stay and the required monitoring.

An important area of research involves mechanically ventilated septic patients. In many basic and translational studies, dexmedetomidine has demonstrated anti-inflammatory and bacterial killing properties superior to those of gamma-aminobutyric acid (GABA) agonists, thereby, reducing the incidence of subsequent infections and 28-day mortality in patients with sepsis (28, 40). Although indirectly correlated, another secondary analysis of the SPICE III trial (41) was carried out in patients with septic shock. On multivariable-adjusted analysis, dexmedetomidine appeared to be associated with lower vasopressor requirements to maintain the target MAP.

Despite the immunomodulatory and anti-inflammatory properties of dexmedetomidine, in a recent multicenter, double-blind, randomized, controlled trial that enrolled adult mechanically ventilated patients with sepsis, the use of dexmedetomidine for light sedation did not demonstrate more days of life without acute brain dysfunction (coma or delirium) compared with propofol. In addition, no differences were found in ventilator-free days at 28 days, death at 90 days, or global cognition at 6 months (42).

## Neuromonitoring to Guide Sedation

As mentioned earlier, light sedation aims to obtain the minimum depth of sedation required to keep the patient free from agitation and anxiety, avoiding oversedation. Traditionally, the most widespread method to monitor the depth of sedation is clinical, using Richmond Agitation Sedation Scale (RASS) or Riker Sedation-Agitation Scale (SAS). However, clinical scales require awake patients and are not useful to monitor deep sedation (43). Therefore, other methods have been developed in the recent times to evaluate sedation depth through processed- and raw-EEG signals. Processed-EEG signal (Bispectral Index, PSI, Entropy) has been proved, both in OR and ICU, to be related to the depth of sedation measured with clinical scales (43, 44) and to reduce sedative dosages (45). Low values of a processed-EEG index (corresponding to unnecessary deep sedation, burst-suppression or isoelectric EEG) are associated with a higher incidence of delirium and mortality (46). Moreover, processed-EEG monitoring systems can also show the raw EEG traces and spectral quantitative array, allowing clinicians to identify specific electroencephalographic signatures of sedative drugs in addition to the general benefits of raw EEG monitoring (47).

Concerning the impact of objective measurement of the level of sedation on outcomes, the literature is scarce on high-quality studies. A recent systematic review and meta-analysis (48) included four RCTs and found no benefits of BIS monitoring on the clinical outcomes or resource utilization. A possible explanation of this conflicting and insufficient evidence may rely on the intrinsic limitations of the numerical dimensionless scales of alertness/unconsciousness derived from the electroencephalographic signal (49–51) rather than the electroencephalogram itself which presents a strong neurobiological background supporting its use (47, 52) which should be promoted by the validated educational programs (53).

## Conclusion

The approach to the management of critically ill patients is multidimensional, and targeted sedation should be considered in the context of the ABCDEF bundle. No doubt exists about the need to limit deep sedation to restricted clinical circumstances, while the light sedation (or non-sedation) approach could not explicit its beneficial effects when taken alone and not in the context of an ABCDEF bundle approach. When light sedation is applied, propofol or dexmedetomidine should be used, carefully evaluating their possible contraindications. Moreover, whenever clinical evaluation of the level of sedation is not feasible, a processed and raw EEG signal could be useful to avoid the detrimental effects of over-sedation (Figure 1).

## Author Contributions

All authors contributed to the literature review, drafting, and critical revision of the manuscript.

## Conflict of Interest

The authors declare that the research was conducted in the absence of any commercial or financial relationships that could be construed as a potential conflict of interest.

## Publisher’s Note

All claims expressed in this article are solely those of the authors and do not necessarily represent those of their affiliated organizations, or those of the publisher, the editors and the reviewers. Any product that may be evaluated in this article, or claim that may be made by its manufacturer, is not guaranteed or endorsed by the publisher.
